# 电磁导航支气管镜在胸外科中的应用及进展

**DOI:** 10.3779/j.issn.1009-3419.2022.101.02

**Published:** 2022-02-20

**Authors:** 超 郭, 夏尧 刁, 诚 黄, 野野 陈, 晔 张, 单青 李

**Affiliations:** 100730 北京，中国医学科学院北京协和医学院，北京协和医院胸外科 Department of Thoracic Surgery, Peking Union Medical College Hospital, Chinese Academy of Medical Sciences and Peking Union Medical College, Beijing 100730, China

**Keywords:** 肺周围小结节, 电磁导航支气管镜, 一体化杂交手术室, Small pulmonary peripheral lesions, Electromagnetic navigation bronchoscopy, Hybrid operating room

## Abstract

肺癌是目前我国癌症发病率和死亡率最高的肿瘤。随着影像学技术的发展与普及，肺周围小结节（small pulmonary peripheral nodules, SPPNs）的检出率越来越高，而SPPNs的精确定位及定性一直是胸外科临床诊治过程中的难题。电磁导航支气管镜（electromagnetic navigation bronchoscopy, ENB）的问世，为肺外周病灶的诊治提供了新的微创手段。本文将对ENB在外科手术前定位、诊断和局部治疗中的应用及进展，以及ENB在一体化杂交手术室的临床应用情况进行综述。

## Abstract (en)

Lung cancer ranks the first cancer-related morbidity and mortality in China. With the development and penetration of imaging technology, increasing small pulmonary peripheral Nodules (SPPNs) have been detected. However, precise location and diagnosis of SPPNs is still a tough problem for clinical diagnosis and treatment in department of thoracic surgery. With the development of electromagnetic navigation bronchoscopy (ENB), it provides a novel minimally invasive method for the diagnosis and treatment of SPPNs. In this review, we summarized the application and progress of ENB in preoperative positioning, diagnosis, and local treatment, then, discussed the clinical application of ENB in the hybrid operating room.

肺癌是国内乃至世界范围内最常见的恶性肿瘤之一，根据2015年中国癌症统计数据^[1, 2]^，中国肺癌的发病率和死亡率均排名第一。近年来，随着低剂量电子计算机断层扫描（low-dose computed tomography, LDCT）在肺癌早期筛查中的应用，肺癌的早期检出率提高了12%，病死率降低了20%^[3, 4]^。与此同时，肺周围小结节（small pulmonary peripheral nodules, SPPNs）的检出率越来越高，但是其中大多数结节很难从影像上确定其良恶性。在如此多的结节中识别出需要手术干预的早期肺癌是临床诊断治疗中的难题，目前临床上常用的技术对SPPNs的诊断仍存在准确率较低、创伤过大或可能出现严重并发症等问题^[5-7]^。尤其是对于磨玻璃结节（ground glass nodules, GGN）这样的病变，很少有实性成分，术者难以通过胸腔镜辅助手术过程中传统的手指触摸感知或者器械辅助来识别结节的位置，这给外科医生尤其是年轻医生带来了巨大挑战^[8]^，有报道传统方法定位失败进而中转开胸的比例高达54%^[9, 10]^。这就为临床上诊治肺小结节疾病提出了新的需求。电磁导航支气管镜（electromagnetic navigation bronchoscopy, ENB）最早于21世纪初进入临床实践应用，其利用电磁传感器，同时结合计算机虚拟支气管镜与高分辨率螺旋CT的特点，既可准确到达传统支气管镜无法到达的肺外周病灶实时导航定位，又可获取病变组织进行病理活检，并可以直接进行消融等介入治疗^[11]^。ENB技术的出现为肺外周小结节提供了更加精确的定位、定性手段，可与微创胸外科优化融合，仅通过一次麻醉即可完成肺部病灶的“诊断、定位、手术”一体化诊疗模式，满足肺癌早发现早诊断早治疗的临床需求。因此由胸外科主导的肺外周结节优化诊疗流程，使诊疗具有更好的连续性，本研究综述ENB在胸外科中的应用进展。

## 1 外科手术前定位

难以触及的SPPN的术中定位是世界范围内胸外科的难题，目前常采用的是CT引导下经皮染料注射或者穿刺钩针、弹簧圈定位等方法，然而CT引导下这些定位方法需要患者定位后限制活动，否则容易脱落而造成手术探查失败，再者易引起气胸、穿刺部位疼痛及肺实质出血等风险。ENB的出现为外科医生提供了新的定位方法，与传统的经皮穿刺定位方法相比，通过ENB进行染料/造影剂注射定位肺小肺结节可以显著减少染料扩散和气胸的机会，因为通常ENB操作不会破坏胸膜，从而提高外科手术的安全性和准确性^[6]^。

### 1.1 局部染料注射

已经有多个研究小组报告了用亚甲蓝或靛蓝胭脂红混合不同溶媒通过ENB进行肺小结节的术前定位标记，随后成功进行胸腔镜肺切除手术，有效染色可持续达120 min，研究结果显示ENB相关并发症的发生率可忽略不计^[4, 12]^。在这些研究中，ENB染料定位成功率从79%-100%不等，部分研究者认为定位染色失败主要是因为染料渗入胸膜腔或肺实质，或者长期吸烟患者肺膜表面黑色素沉积影响边界判断^[13-15]^。有趣的是，结节直径和距离胸膜表面的距离似乎并没有影响肿瘤的定位及灵敏度^[16, 17]^。为了尽可能减少染料外染的可能性，多项研究探索技术的改进，如果结节位于胸膜附近，则将小剂量的亚甲蓝（0.5 mL-1 mL）直接注入病变处。对于距离胸膜5 mm-10 mm以上的那些小结节，可以在病变和最近的胸膜表面中途释放染料，或者可以采用双重染料释放方法（在病变处和胸膜表面）^[18]^。Qian等^[8]^尝试在结节部位注射染料剂量为0.8 mL/cm（病变直径），随后通过ENB定位鞘往复运动（Massage）进行局部脏层胸膜的染色。Luo等^[15]^发明了一种通过将亚甲蓝与纤维蛋白密封剂混合来控制染料位置的新方法，除了增加可视化的染色，还增加了凝胶状的触感。对于相对复杂的定位，可以在结节周围3处-4处位置进行染色，从而勾画出结节所在范围。

### 1.2 荧光镜技术

根据Anayama等先前的研究^[19]^，近红外荧光胸腔镜可以检测到距充气肺表面最深24 mm处的吲哚菁绿（indocyanine green, ICG）发出的荧光。在其研究团队最新报道中，通过ENB注射ICG随后通过近红外胸腔镜检查成功率为95.5%（21/22），同时也可以用于多处病灶的标记^[20]^。He等^[21]^报道了将ENB技术应用于不插管剑突下荧光胸腔镜双侧肺楔形切除的术前定位，通过ENB将ICG注射于双侧肺结节位置，随后通过荧光胸腔镜视野定位结节，成功进行楔形切除。通过此种方式将手术创伤尽可能的降低，手术实施的更加精准。

### 1.3 矢量定位法

我国学者矫文捷手术团队^[22]^报道的ENB矢量定位法通过ENB导航后变换患者体位为健侧卧位，助手轻推导航定位装置使脏层胸膜表面形成隆起，成功为22例患者的肺外周结节进行定位及胸腔镜下楔形切除治疗（22/22, 100%），结节平均大小为（11.0±3.6）mm，距离脏层胸膜表面距离为（16.5±6.2）mm，定位时间平均为（17.5±4.2）min，认为该方法安全可行有效，可作为可选的一种定位方法。

### 1.4 局部放置标记物

另外多项研究报道了经ENB引导放置弹簧圈等基准标记物，既增加治疗过程中的定位准确性，也降低了气胸等并发症发生率^[23-25]^。

## 2 术前诊断

越来越多的临床研究证实，通过ENB可对SPPN活检取样，对胸外科术前明确病理诊断具有重要价值。2006年Gildea等^[26]^发表了在美国开展的首次大规模前瞻性ENB临床研究，ENB引导下经支气管肺部病灶取样成功率为74%（40/54），恶性病变确诊率为74.4%（32/43）。Gex等^[27]^在2014年对ENB诊断肺外周小结节进行了一项系统回顾和*meta*分析，回顾了15项临床研究（1, 033个病灶），诊断阳性率为64.9%，准确率为73.9%，肺癌诊断的灵敏度为71.1%，阴性预测值为52.1%，并发症气胸发生率为3.1%，1.6%需要胸腔闭式引流，结节位于中上肺、结节大小、注册准确性、CT提示支气管征、联合使用超声探头、合理利用抽吸技术可以提高准确性。2015年由Folch等^[28]^开始的全球多中心前瞻性队列研究NAVIGATE（Clinical Evaluation of super Dimension^TM^ Navigation System for Electromagnetic Navigation Bronchoscopy），统计了在全球29个医学中心诊治的1, 215个病例，结节平均大小为20.0 mm，91%以上的患者结合使用了术中X线验证，平均系统注册、计划时间5 min，ENB操作时间25 min，94%（1, 092/1, 157）成功获得了病理，恶性占44%，敏感性、特异性、阳性预测值和阴性预测值分别为69%、100%、100%和56%，而并发症发生率气胸为2.9%，血胸为1.5%，呼吸衰竭为0.7%。根据目前数十项ENB相关研究来看，其诊断率分布在33%-97%，大部分报道为67%-84%，波动范围较大，这与操作者经验、ENB的精准性、有无联合支气管内超声（endobronchial ultrasound, EBUS）、SPPN位置和大小、有无支气管征等有关^[27, 29-33]^。Seijo等^[32]^发现存在支气管征患者的ENB诊断率明显高于无支气管征患者（79% *vs* 31%）。Eberhardt等^[34]^通过前瞻性随机对照研究结果发现，联合应用ENB和径向EBUS可提高诊断准确率（88%），明显高于单独使用ENB组（59%）和径向EBUS组（69%），分析原因为ENB可实时导航，而径向EBUS使病灶可视化，从而实时确认，两者结合促使ENB更精准有效。针对ENB精准程度受肺部呼吸运动影响，Flenaugh等^[35]^分享了一套ENB系统，其纳入吸气相/呼气相的CT图像，并追踪操作过程中的呼吸运动，确保操作过程中动态校准，降低呼吸对结节定位的干扰；升级活检工具也带电磁定位，确保整个过程实时跟踪，避免盲目取样。Deng等研究者^[36]^通过对相关研究的汇总分析回顾对比了不同的SPPN诊断方式的进展，其中包括CT引导经皮肺穿刺活检相关文献22篇，ENB相关文献31篇，经气管镜超声引导针吸活检相关文献66篇，纵隔镜相关文献15篇，循环肿瘤细胞检测相关文献19篇。其中，ENB检出率为79.79%±15.34%，最常见的并发症为气胸，发生率为5.2%（86/1648）。CT引导经皮肺穿刺活检的敏感性和特异性为92.52%±3.14%和97.98%±3.28%，但其并发症气胸（22.69%）和血胸（7.08%）发生率较高。最终Deng等认为应该根据SPPN的位置和性质来选择适合的活检方式，当处理更小、肺实质内更深的结节时，ENB更具优势。

## 3 治疗

目前临床上诊治的多原发肺癌逐渐增多，通常无法通过手术切除所有病灶，局部治疗是一种必要的补充治疗方式。之前的多项研究表明，基于介入或内镜的治疗方法，例如近距离放疗、射频消融（radio-frequency ablation, RFA），微波消融，冷冻消融和光动力疗法（photodynamic therapy, PDT）等是安全可行的，其并发症是尚可接受的。此外，经支气管导管直接输送化疗药物或免疫治疗药物到病变部位也是ENB的一个重要治疗用途。ENB的临床应用为肺癌患者的局部治疗提供了新的方向。

### 3.1 局部放疗

Harms等^[37]^在2006年报道了通过ENB和超声确认肺部病变，沿EWC在病变处插入6 -F放疗导管后通过铱192进行近距离放疗。回顾18例患者结果表明，50%（9/18）的患者肿瘤治疗疗效达到完全缓解，另外50%（9/18）患者取得部分缓解，未出现明显不良反应。证实ENB引导的近距离放疗对不可切除的周围型肺癌是安全有效的方法，可减少对周围正常组织的损伤。

### 3.2 射频消融

由于部分患者存在合并症或禁忌症而不适宜行手术治疗，RFA作为一种局部治疗，可使不能接受手术的患者拥有与接受了亚肺叶切除或放疗的患者相似的总生存率^[38, 39]^。从最开始Tsushima等^[40]^探索经支气管对绵羊肺进行射频消融治疗的可行性，到近年来，有研究^[41]^报道了通过融合ENB和RFA技术治疗了2例被诊断为Ia期肺癌和1例被诊断为肺转移的患者，随后对这3例患者进行随访，除了其中1例患者在随访的6个月后出现进展，另2例均获得1年的无进展生存期。

### 3.3 微波消融

近年来，随着ENB技术的成熟，匹配ENB的经支气管微波消融技术也逐渐成熟，2008年起，Wolf等^[42]^将微波消融开始用于治疗早期肺癌（*n*=50），证实了其安全性。柔软的微波消融针，更能适应肺部错综复杂的支气管结构；结合水循环冷却技术，使输出功率达到足以灭活一定范围内的肿瘤细胞，即可局部治疗甚至治愈肺癌。目前文献对于经支气管微波消融治疗肺周围小病变（small peripheral lung lesions, SPLL）的报道则较少。上海胸科医院2016年5月成功施行国内首例ENB精确引导下经支气管微波消融术，随后多家单位开始不断尝试调整功率与时间的不同组合，试图摸索出最佳的消融模式。Jiang等^[43]^报道了1例双肺多原发肺癌患者，根据术前计划，先通过ENB活检右肺上叶纯磨玻璃病灶，证实为早期腺癌后通过ENB引导微波消融行局部治疗，而后对左侧多发肺部结节行常规胸腔镜手术治疗，患者术后顺利康复出院，治疗效果待随访，为多原发肺癌的诊治提供了新策略。

### 3.4 光动力治疗

也有学者探索将ENB同光动力治疗相结合。在Chen等^[44]^的研究中，通过ENB成功地对3个平均大小为21.3 mm的肺结节进行了PDT。在随访CT扫描中，所有患者的肿瘤均明显缩小（包括2例部分缓解和1例完全缓解）。关于治疗相关并发症，PDT后1个月只有1例患者出现皮肤超敏反应。近几年国内已研发成功1, 000 mW高功率氦氖激光肿瘤治疗仪（波长为630 nm），并被国家科技部列为重点研发产品，临床应用也已取得非常好的疗效。

### 3.5 局部药物治疗

无论是基于导管的治疗还是直接将药物递送至肿瘤或病变都不是新概念，但过去往往需要通过经皮途径或支气管镜进行，并且仅限于靠近肺部近端的气道的病变。而ENB的出现使得消融导管和药物输送系统可到达肺部的更外围区域，并使得在单次疗程中治疗多个病变更容易实现，有效地规避了经皮入路的缺点和并发症，尤其是在针对多个病变时^[45]^。

综上，ENB引导下经支气管的局部治疗的有效性、安全性、远期疗效及最佳适应症等仍需更多的前瞻性临床研究和长期随访确认。

## 4 一体化杂交手术室

前期的临床实践表明，要通过支气管镜放置活检工具或进行其他介入治疗时，需要将ENB的扩展工作通道（extended working channel, EWC）固定在朝向目标病变的位置方向^[4]^。目前有报道通过多种技术用于验证ENB期间EWC的方向。在已报道的研究中，通常使用术中X光透视来确认EWC的位置，但某些病变，尤其是直径比较小的磨玻璃结节，使用X射线检查很难发现。当需要同时进行淋巴结活检或处理更具挑战性的病例时，则通常采用超声内镜协助。在NAVIGATE临床试验中，术中X光透视检查、支气管内超声检查和CT检查分别占91%、57.4%和4.9%^[28, 46]^。而影像学检查无论是对ENB的实时辅助还是调整，都需要一体化杂交手术室这一平台的支持。

近年来，一体化杂交手术室在胸外科微创手术中的应用获得广泛认可。当过往不具备杂交手术室时，胸外科医生通常会先让患者接受经皮Hookwire定位或注射染料，随后将患者送入手术室进行手术。相比之下，杂交手术室中的ENB标记技术为胸外科医生提供了更加灵活的手术计划。更为直接的是，在专门设计的杂交手术室内连续进行诊断、定位和手术可以提高诊断准确性，并减少与金属标记物植入相关的并发症风险。当治疗多发肺部病变时，这一点则显得更为重要，精确的肺结节定位技术可以避免正常肺组织被不必要的切除^[11, 45]^。杂交手术室中装备的DynaCT可提供高分辨率的实时图像，这可以改善ENB导航期间的EWC位置确认和进一步提高可视化。在全身麻醉和单腔气管插管后，将患者仰卧，ENB电磁板置于患者下方计划位置，随后将支气管镜置入并到达目标段支气管。基于ENB操作屏幕上的术前CT数据，可通过虚拟3D气道在导航路径下将电磁可定位导向器朝靶病变方向移动。随后进行DynaCT扫描，活检工具与EWC将被锁定并保持不动，从而显示出活检针的精确方向。如果位置不正确，则根据实时CT图像调整活检工具位置。如果需要，可以进行第二轮术中CT扫描，以通过查看矢状和冠状图像来确保活检工具确实在病变内。如果活检病理结果证实需要进行胸腔镜手术，则将单腔气管插管替换为双腔气管插管，并将患者置于满足手术需要的侧卧位。一旦EWC偏离目标病变，反复进行活检/刷涂几乎不会增加ENB的诊断率。因此，在杂交手术室中进行ENB操作的优势在于尽可能的克服了由导航偏差、CT检查误差和活检工具移动所导致的“误击”现象，从而提高了ENB在诊断小病变中的准确性和有效性^[47, 48]^。但值得注意的是，在杂交手术室环境中使用ENB系统时，也可能会出现ENB未能显示适当的路径，此时就需要考虑结合其他定位方法。有研究^[47]^报道，针对不同的肺部结节可以同时进行ENB染色和经皮穿刺Hookwire定位，这就需要在杂交手术室中一站式解决。杂交手术室的便捷工作流程将使染料扩散和Hookwire脱钩的风险降到最低。近期，Cheng及其团队^[49]^报道了一种通过结合ENB和DynaCT两种设备实现对深部肺结节（距离胸膜 > 10 mm）的双重标记定位，双重标记是指通过ENB注射的近红外染料作为表面标记，随后从同一EWC置入微型弹簧圈作为深部标记。此方式既避免了经皮穿刺等有创操作，也通过双重标记提供的实时图像大幅减少了由呼吸造成的干扰。此外，有研究团队^[50]^开发了一种三合一染料标记技术，将碘海醇造影剂、亚甲蓝和ICG混合，通过ENB进行注射定位。可以通过DynaCT扫描实时观察病变处的碘海醇注射范围。在随后的胸腔镜手术中，亚甲蓝可以提示病变的位置。如果发生胸膜粘连或其他无法识别亚甲蓝边界的情况，近红外光胸腔镜则可以将ICG定位为备用策略。还有研究报道了一种智能电子标记，有望取代各种染料标记用于肺结节定位。这些可以发射微小射频的标记物可以通过ENB输送到一个或多个需要手术定位的肺部病变中或其附近^[45]^。

## 5 展望

自20世纪90年代中期ENB问世以来，临床医生不断探索其适宜开展的患者和病变、以及影响ENB性能的技术因素。随着ENB临床应用广泛开展，不断证实其安全性、有效性，同时ENB经自然腔道微创、无辐射伤害等优点，与其他技术优化融合，仅通过一次麻醉即可完成SPPN的“诊断、定位、手术/局部治疗”一体化诊疗模式，满足肺癌早发现早诊断早治疗的临床需求，未来有可能改变肺癌的诊断和治疗方式。但不得不承认，ENB仍存在一定的局限性，对SPPN的诊断率仍不能令人满意，定位的精准度受到支气管镜技术的制约，定位操作步骤相对繁琐，此外检查成本相对昂贵，限制了其在临床上广泛普及。呼吸运动补偿、移位飘移等问题需要进一步研究探索。展望未来，全程引导、SPPN位置的实时确认和可视化、实时跟踪活检工具、开发更先进的治疗工具、机器人技术和成像技术等仍是未来ENB临床应用需要解决的难题，从而使ENB引导下的诊断和治疗更精准、更有效。
